# Complementary and alternative therapies for stable angina pectoris of coronary heart disease: A protocol for systematic review and network meta-analysis

**DOI:** 10.1097/MD.0000000000028850

**Published:** 2022-02-18

**Authors:** Guanyu Wang, Feiran Li, Xu Hou

**Affiliations:** a Department of health care, Huaiyin people's Hospital, Jinan City, Shandong Province,China; b First College of Clinical Medicine, Shandong University of Traditional Chinese Medicine, Jinan City, Shandong Province, China; c Department of Endocrinology and Metabolic Diseases, Shandong Provincial Hospital Affiliated to Shandong First Medical University, Jinan City, Shandong Province, China.

**Keywords:** complementary and alternative therapies, coronary heart disease, protocol, stable angina pectoris, web meta-analysis

## Abstract

**Background:**

Stable angina pectoris in patients with coronary heart disease is a clinical syndrome of rapid transient ischemia and hypoxia of myocardium due to the increase of myocardial load on the basis of fixed severe coronary artery stenosis. In recent years, the incidence rate of this disease has been rising steadily, which seriously threatens human life and health. When the disease occurs, its complementary and alternative therapy can relieve chest pain, improve cardiac function, and reduce adverse events. However, in retrospect of all the studies, we lack systematic analysis of the efficacy and safety of various complementary and alternative therapies. The curative effects were ranked. On the basis of these findings, we conducted a study of complementary and alternative therapy in patients with coronary heart disease, and proposed a network meta-analysis (NMA) protocol to explore the efficacy of different complementary and alternative therapies.

**Methods:**

We will comprehensively search the Chinese and English databases from the creation date to January 15, 2022. The randomized controlled trials of the supplementary and alternative treatment of stable angina pediatrics in patients with coronary heart disease and the relevant literature of the ongoing trials will be published. The 2 researchers will conduct literature screening and data extraction independently, using Cochrane system evaluator manual 5 3. The recommended bias risk assessment tool was used to evaluate the quality of the included study, Q-test was used and combined with heterogeneity analysis, and the analysis sensitivity was observed. The Review Manager 5.4 software provided by Cochrane Collaboration Network is used to statistically analyze the included literature, and the obtained results are made into forest map and funnel map for data analysis and processing. It is suggested that the evaluation will be used to formulate and evaluate the level, so as to classify the quality of NMA evidence.

**Results:**

Through analysis, we will get the efficacy and safety ranking of different complementary and alternative therapies in the treatment of stable angina pediatrics in patients with coronary heart disease, so as to provide further reference for the selection of clinical treatment methods.

**Conclusion:**

The complementary and alternative treatment of stable angina peptis in patients with coronary heart disease has a positive effect on improving its symptoms. This study can provide evidence support for clinicians and patients.

**INPLASY registration number:**

INPLASY202210066.

## 1 Introduction

Coronary atherosclerotic heart disease (CHD) refers to heart disease caused by luminal stenosis or occlusion due to atherosclerotic lesions occurring in the coronary arteries, which leads to myocardial ischemia and hypoxia,^[1]^ and in recent years, with the continuous adjustment of dietary structure and lifestyle, as well as an increasing aging population, its morbidity and mortality are increasing, Moreover, the age of onset is younger, and due to its increasing prevalence and death, it causes a huge economic burden, and CHD has become a major public health problem worldwide.^[^2^–^4^]^

CHD can be divided into 2 main groups: chronic coronary diseases and acute coronary syndromes. The former includes stable angina, ischemic cardiomyopathy, and covert cardiomyopathy, and the latter includes unstable angina, non-ST segment elevation myocardial infarction, and ST segment elevation myocardial infarction.^[5]^ Angina, a clinical syndrome characterized primarily by chest pain due to transient myocardial ischemia, is the most common manifestation of CHD. Coronary stable angina, also known as exertional angina, is a clinical syndrome of transient ischemic hypoxia in the myocardium acutely due to an increase in myocardial load on the basis of a fixed, severe stenosis of the coronary arteries. It is characterized by paroxysmal anterior chest pressor pain or a feeling of distress that is mainly located in the posterior part of the sternum and may radiate to the precordial area or ulnar aspect of the left upper extremity. It often occurs when there is an increase in exertional load, which can last for several minutes, with the disappearance of pain at rest or after the use of nitrate preparations.^[6]^ The extent, frequency, and nature of the pain paroxysms, as well as the precipitating factors, can be remarkably unchanged for weeks to months. If there is a change, it can occur as unstable angina. In recent years, the incidence of stable angina pectoris (SAP) in patients with West coronary heart disease (CHD) has been increasing, and some reports have indicated that SAP in patients with West CHD during development is accompanied by a certain degree of autonomic dysfunction, not only easy to induce but also increase the risk of developing cardiac autonomic dysfunction. Meanwhile, the cardiac function stability was reduced to some extent. Stable angina is not only a key factor affecting the quality of life of patients, but is also tightly associated with the long-term prognosis of patients.^[7]^

At present, the methods of Western medicine for the treatment of SAP in patients with West CHD are mostly aimed at relieving the symptoms of angina pectoris and improving the quality of life of patients, such as long-term oral antiplatelet drugs, stable plaque drugs, and coronary vasodilator drugs.^[8]^ If the above drugs do not work well, cardiac coronary CT or cardiac coronary angiography may also be performed to observe the cardiac vasculopathy. These treatments have a role in improving clinical symptoms and long-term outcomes, but they also have some difficult problems, such as angina that persists after intervention, adverse effects caused by therapeutic drugs that paradoxically increase the risk of other adverse events, and so on. Integrated traditional and Western medicine in the pattern of disease syndrome provides a new idea and method for prevention and treatment, which increasingly receives international attention, and shows a high advantage of action.

At the onset of CHD-SAP, its complementary and alternative therapies are able to relieve chest pain symptoms, improve cardiac function, and reduce the occurrence of adverse events, etc, generally. Chinese herbal medicines such as rapid rescuing heart pill, Salvia miltiorrhiza dripping pill, and muskbouxin pill can be selected, and TCM syndrome differentiation is based on the syndrome. Acupuncture, acupoint application, Tuina, and traditional utilitarian as non-drug therapies in traditional Chinese medicine (TCM) have a certain effect on relieving angina symptoms and improving myocardial ischemia,^[9]^ which can play a certain role in decreasing the number of angina attacks and delaying the progression of the disease in patients with CHD-SAP under the guidance of TCM theory and rational prevention of perturbation methods. However, reviewing all current studies, we found a lack of systematic analysis of the efficacy and safety of various complementary and alternative therapies, ranking their efficacy. On the basis of these findings, we conducted a study of complementary and alternative therapies in patients with SAP in CHD and proposed a network meta-analysis (NMA) protocol to explore the efficacy of different complementary and alternative therapies.

## 2 Method

In this study, we will use Bayesian network meta-analysis and NMA. According to the PRISMA 2020 (preferred reporting items for systematic reviews and meta-analyses) PRISMA statement, a literature review was conducted.^[10]^

### 2.1 Study registration

The protocol of this NMA has been registered on the international platform for registration system evaluation and meta-analysis protocol (inpasy). The registration number is # implasy202210066 (URL: https://inplasy.com/inplasy-2022-1-0066/).

### 2.2 Inclusion criteria

#### 2.2.1 Type of study

We will include randomized controlled trials (RCTs) of complementary and alternative therapies related to this study published in China and internationally. The language is limited to Chinese and English.

#### 2.2.2 Participants

Patients diagnosed with sap-chd. Subjects were required to meet the following criteria: onset of symptoms such as SAP, coronary heart disease-related dyspnea; Previously symptomatic, on medication, or asymptomatic after revascularization; Microangiopathy; Vasospasm. Age, sex, race, or nationality were not considered.

#### 2.2.3 Intervention and comparison

Chinese herbal compound, Chinese patent medicine, acupuncture, acupoint application, Tuina, extirpation tank, traditional utilitarian methods, etc, were used in the experimental group. The above various treatments can be used alone or in combination. The control group was given conventional treatments, including western medicine, placebo, and no treatment.

#### 2.2.4 Results

Main results: angina clinical symptoms, electrocardiographic effects, efficacy of Chinese medical syndromes, nitroglycerin withdrawal rate, exercise treadmill test.

Secondary results: EF value, 6-min walk test, levels of inflammatory factors, improvement of endothelial function, and lipid changes.

### 2.3 Exclusion criteria

Non-RCT studies, reviews, secondary studies, conference proceedings, duplicate published literature; Studies with incomplete and unavailable data reporting; Other studies such as animal experiments.

### 2.4 Search strategy

Between December 2019 and November 2021, we will comprehensively search the following databases for relevant literature published from their creation dates to January 15, 2022: the Chinese biomedical literature database (CBM), China National Knowledge Infrastructure (CNKI), Wanfang database (Wangfang)VIP database (VIP), PubMed, EMBASE database, Cochrane Library, Central Register of clinical trials, and Government clinical registration system. Limited to reports published in Chinese and English. The language is limited to Chinese and English. Retrieval skills and considerations will be studied in detail, and the final retrieval strategy will be determined after multiple searches. The search strategy will be constructed in the form of medical subject headings (mesh) combined with synonyms and combined with keywords, including stable angina, stable angina, angina, coronary atherosclerotic heart disease, coronary artery disease, coronary atherosclerosis, thoracic paralysis, heart pain, chest pain, traditional Chinese medicine, traditional Chinese medicine, acupuncture, Tuina, and so on. The search was expanded to include references retrieved and reviewed according to the above keywords. The titles and abstracts of the retrieved literatures were reviewed by each of the 2 investigators in this study group to determine whether they were included in the analysis according to the criteria. To avoid double counting of study subjects who were included by more than 1 literature, corresponding authors were also contacted for clarity if necessary. If a group of study subjects appeared to be included by more than 1 piece of literature, data were extracted from the recently published literature. For literatures that were uncertain whether the criteria were met, the full texts were downloaded for further evaluation and decided by CO discussion. Database search PubMed was used as an example, and the search strategy is summarized in Table 1.

**Table 1:** Search strategy for PubMed.

NO.	Search item
#1	“Coronary heart disease ” [MeSH Terms]
#2	“Coronary heart disease ”[Title/Abstract] OR ”Coronary atherosclerotic heart disease”[Title/Abstract] OR ”coronary disease”[Title/Abstract] OR ”atherosclerosis”[Title/Abstract] OR “CAD”[Title/Abstract] OR”” CHD[Title/Abstract]
#3	#1 OR #2
#4	“stable angina pectoris” [MeSH Terms]
#5	“stable angina pectoris”[Title/Abstract] OR “ stable angina”[Title/Abstract] OR “stable anginas“[Title/Abstract] OR “chronic stable angina” [Title/Abstract] OR” angina pectoris”[Title/Abstract]
#6	#4 OR #5
#7	Complementary Therapiesl[MeSH Terms]
#8	alternative therapies[Title/Abstract] OR Therapies, Complementary[Title/Abstract] OR Therapy, Complementary[Title/Abstract]OR”ComplementaryMedicine[Title/Abstract]OR Medicine,Complementary[Title/Abstract] OR Alternative Medicine[Title/Abstract] OR Medicine, Alternative[Title/Abstract] ORAlternative Therapies [Title/Abstract] ORTherapies, Alternative[Title/Abstract] OR Therapy, Alternative[Title/Abstract]
#9	#7 OR #8
#10	Place-bo[Title/Abstract] OR Chinese medicine[Title/Abstract] OR traditional Chinese medicine[Title/Abstract] OR acupuncture[Title/Abstract] OR tuina[Title/Abstract] OR moxibustion[Title/Abstract]
#11	#9 OR #10
#12	Randomized controlled[Publication Type] OR Controlled Clinical Trial[Publication Type] OR Randomized[Title/Abstract] OR Randomly [Title/Abstract] OR random allocation[Title/Abstract]
#13	#3 AND #6 AND #11 AND #12

### 2.5 Study selection and data extraction

Literature screening and data extraction were performed independently by 2 investigators. Duplicates were identified by Noteexpress and hand checked; According to the established inclusion and exclusion criteria, the literature title and abstract were read, and those that obviously did not meet the inclusion criteria were excluded; then, the literature was subjected to full-text acquisition and reading, and those that did not meet the inclusion criteria or had reasons for exclusion were further excluded, and the results were cross checked, if disagreements were resolved by discussion or consultation with a third party.

Data extraction for included studies was performed separately by 2 investigators using a prespecified data extraction sheet, which included the following: basic information of patients, baseline condition of patients’ diseases, interventions/control measures and their courses, outcome measures; Results were cross checked, and disagreements were resolved by discussion or consultation with a third party.

### 2.6 Risk of bias assessment quality

The Cochrane Handbook for systematic reviewers was used by 2 investigators separately. 5 3 the recommended risk of bias assessment tool evaluates the quality of included studies in terms of 7 aspects: randomization method, allocation concealment, blinding of subjects, blinding of outcome assessment, data integrity, selective reporting, other bias (e.g., whether the included trials reported the funding source, conflict of interest, baseline circumstances, etc). For each entry, the method use was correctly classified as low risk (low risk), the method use description was unclear as unclear risk (unclear risk), and the method use was incorrect or not used as high risk (high risk). This was done independently by the investigators and cross checked, and in case of disagreement was resolved by discussion or assisted by a third investigator.

### 2.7 Heterogeneity test

Using Q test combined with heterogeneity analysis, when *P* ≥ .10, there was no heterogeneity or heterogeneity among different studies that could be ignored. When *P* < .10, there was heterogeneity among different studies, and heterogeneity could be ignored when < 0.4; When 0.4 ≤ ≤ 0.6, there was moderate heterogeneity among different studies; > At 0.6, the heterogeneity among different studies was high. When heterogeneity was high, single studies were removed (setting weight = 0%), sources of heterogeneity were discussed, and sensitivity was observed.

### 2.8 Data analysis

#### 2.8.1 Statistical analysis

Review Manager 5.4 software provided by the Cochrane Collaboration was applied to summarize the included literatures statistical analysis, and the results obtained were subjected to forest and funnel plots. The odds ratio (or) was used for count data, the weighted mean difference (WMD) was used for continuous variables as the effect scale, and the standardized mean difference (SMD) was applied for count data. Each effect size was expressed with 95% confidence intervals (CIs), and *P* < .05 was considered statistically significant.

#### 2.8.2 Sensitivity analyses

Sensitivity analysis refers to factors that influence the outcome of a study by changing the inclusion criteria of the literature, for example, randomization, loss to follow-up or withdrawal, different statistical methods, adjudication criteria for efficacy, and choice of effect size (e.g., odds ratio or relative risk) were used to see the homogeneity between studies or whether the composite final results could be changed and thus determine whether the results were stable. If the results of the sensitivity analysis are the same or have similarities with the results of this meta-analysis, it indicates that the results of the studies have a reliable letter; If the results of the sensitivity analysis differed greatly from those of the present meta-analysis, it would indicate that there are underlying factors in the meta-analysis that have an impact on the efficacy of the interventions.

#### 2.8.3 Interpretation of meta-analysis results

On the basis of the results of this meta-analysis, combined with the grading of the Jadad scale quality scores in the literature, the results of SEN Forest plots (meta-analysis results) were analyzed and interpreted one by one, and funnel plots and sensitivity analyses were performed for representative research items to verify the stability and reliability of the meta-analysis results in this article.

### 2.9 Quality of evidence assessment

We will assess the quality of evidence using the grading of recommendations assessment development and evaluation (grade) tool in 4 levels: high, moderate, low, and very low.^[11]^

### 2.10 Ethics and dissemination

As the data of this study were all from the literature and did not involve any personal privacy, no ethical approval was required. The results of this study will be presented in a peer journal or at a conference.

## 3 Discussion

Stable angina pectoris in patients with CHD modern medical research shows that it is the clinical syndrome of acute, temporary ischemic hypoxia in the myocardium. Western medicine is therapeutically based on lipid lowering, antiplatelet aggregation, and other methods, but its long-term application has some side effects such as damaging liver function, reducing the number of platelets, and increasing the risk of bleeding. Through research, it has been found that TCM has no obvious toxic side effects in treatment, and the clinical efficacy is significant. TCM is an effective adjunctive treatment, and clinical treatment mostly starts from deficiency, stasis, Yu, and phlegm, which are treated through the syndrome differentiation theory of TCM and is individualized by combining the characteristics of patient's body and condition as well as climate.^[12]^ Because Chinese patent medicines generally have the advantages of mild drug resistance and small side effects, it has become more widely used in the clinical application of the treatment of SAP in patients with CHD in recent years. Among them, there is no shortage of drugs with better efficacy than Western medicines, so many Chinese patent medicines have gained the recognition from a wide range of patients, such as rapid acting Jiexin pill, Fufang Danshen dripping pill. Compound Danshen dripping pill is currently one of a small number of Chinese patent medicines that have passed the US FDA clinical drug application (ind), and its pharmacological studies have shown that Danshen and Panax notoginseng, the main components of this medicine, have calcium antagonistic and antioxidant effects and can effectively treat sap-chd. It is one of the Chinese patent medicines recommended by the expert consensus of TCM diagnosis and treatment of SAP in CHD. Nonpharmacological treatments mainly include psychotherapy, behavioral therapy, physiotherapy, acupuncture, usually in combination with other pharmacological treatments, whose efficacy has been demonstrated.^[^13^–^15^]^ Acupoint sticking method is composed of a dosage form including plasters, patches, and dispersions, which mainly consist of activating blood circulation and eliminating blood stasis, activating qi and relieving drugs, and aromatizing invigorating drugs. Acupuncture therapy can not only alleviate the suffering of patients and obtain quite obvious results, but also has a simple operation and fewer adverse side reactions. Many studies in recent years have shown that TCM, as a complementary and alternative therapy, has many advantages in the treatment of SAP in patients with away CHD, such as exact efficacy and little side effects, and its clinical therapeutic effect is better than that of the conventional treatment of Western medicine alone. However, current studies on the effectiveness of TCM on the treatment mechanism and treatment effect of this disease still have many deficiencies, such as a lack of epidemiological investigation studies and basic experimental studies on TCM treatment of this disease. TCM ingredients are more complex, and the study of the onset ingredients in treatment is more difficult than that of the auxiliary components. TCM theory lacks modern scientific theoretical support, has a self-made system, and has difficulties in integrating with modern western medicine. Meanwhile, there is often a lack of scientific and effective controlled studies on the therapeutic effects of TCM, and some research methods are poorly designed or even flawed, leading to the validity of the therapeutic effects of TCM often being questioned. NMA can compare and rank various interventions to assess the efficacy and safety of different complementary and alternative therapies. This study performed an extensive literature search and used a Bayesian model to evaluate complementary and alternative therapies for SAP in patients with CHD, but several inevitable limitations remain. To confirm the efficacy and scientific effectiveness of complementary and alternative therapies in the treatment of SAP in patients with West CHD using a uniform scientific, rigorous, and systematic study that comprehensively considers the effects of various factors and a large sample of controls is a much-needed issue we next need to address.

## Author contributions

**Conceptualization:** Guanyu Wang, Feiran Li.

**Data curation:** Guanyu Wang, Feiran Li.

**Formal analysis:** Guanyu Wang.

**Funding acquisition:** Xu Hou.

**Methodology:** Guanyu Wang.

**Project administration:** guanyu wang, Feiran Li.

**Writing – original draft:** Guanyu Wang.

**Writing – review & editing:** Guanyu Wang.

## References

[1] Tian Y Deng P Li B. Treatment models of cardiac rehabilitation in patients with coronary heart disease and related factors affecting patient compliance. Rev Cardiovasc Med 2019;20:27–33. 31184093 10.31083/j.rcm.2019.01.53

[2] Katta N Loethen T Lavie CJ Alpert MA. Obesity and coronary heart disease: epidemiology, pathology, and coronary artery imaging. Curr Probl Cardiol 2021;46:100655. 32843206 10.1016/j.cpcardiol.2020.100655

[3] Stewart RAH Held C Krug-Gourley S. Cardiovascular and lifestyle risk factors and cognitive function in patients with stable coronary heart disease. J Am Heart Assoc 2019;8:e010641. 30897999 10.1161/JAHA.118.010641 PMC6509727

[4] Di Angelantonio E Thompson A Wensley F Danesh J. Coronary heart disease. IARC Sci Publ 2011;363–86. 22997872

[5] Jespersen L Hvelplund A Abildstrøm SZ. Stable angina pectoris with no obstructive coronary artery disease is associated with increased risks of major adverse cardiovascular events. Eur Heart J 2012;33:734–44. 21911339 10.1093/eurheartj/ehr331

[6] Rousan TA Mathew ST Thadani U. Drug therapy for stable angina pectoris. Drugs 2017;77:265–84. 28120185 10.1007/s40265-017-0691-7

[7] Brainin P Hoffmann S Fritz-Hansen T, et al. Usefulness of postsystolic shortening to diagnose coronary artery disease and predict future cardiovascular events in stable angina pectoris. J Am Soc Echocardiogr 2018;31:870–9.e3. 30077186 10.1016/j.echo.2018.05.007

[8] Valgimigli M Biscaglia S. Stable angina pectoris. Curr Atheroscler Rep 2014;16:422. 24838374 10.1007/s11883-014-0422-4

[9] Yang G He HQ Chen G Wang J. [Effect of traditional Chinese medicine in attenuating coronary heart disease and main risk factors by regulating gut micro-biota]. Zhongguo Zhong Yao Za Zhi 2020;45:29–36. 32237408 10.19540/j.cnki.cjcmm.20190505.502

[10] Shamseer L Moher D Clarke M. PRISMA-P GroupPreferredreporting items for systematic review and meta-analysis protocols (PRISMA-P) 2015: elaboration and explanation. BMJ 2015;350:g7647. 25555855 10.1136/bmj.g7647

[11] Guyatt G Oxman AD Akl EA. GRADE guidelines: 1. Introduction-GRADE evidence profiles and summary of findings tables. J Clin Epidemiol 2011;64:383–94. 21195583 10.1016/j.jclinepi.2010.04.026

[12] Zhang KJ Zheng Q Zhu PC. Traditional Chinese medicine for coronary heart disease: clinical evidence and possible mechanisms. Front Pharmacol 2019;10:844. 31427964 10.3389/fphar.2019.00844 PMC6688122

[13] Layne K Ferro A. Traditional Chinese medicines in the management of cardiovascular diseases: a comprehensive systematic review. Br J Clin Pharmacol 2017;83:20–32. 27195823 10.1111/bcp.13013 PMC5338138

[14] Qiu Y Xu H Shi D. Traditional Chinese herbal products for coronary heart disease: an overview of cochrane reviews. Evid Based Complement Alternat Med 2012;2012:417387. 22536282 10.1155/2012/417387 PMC3320001

[15] Guo N Wang P Yang J. Serum metabolomic analysis of coronary heart disease patients with stable angina pectoris subtyped by traditional Chinese medicine diagnostics reveals biomarkers relevant to personalized treatments. Front Pharmacol 2021;12:664320. 34194326 10.3389/fphar.2021.664320 PMC8236985

